# Genome-wide transcriptome analysis of genes involved in flavonoid biosynthesis between red and white strains of *Magnolia sprengeri* pamp

**DOI:** 10.1186/1471-2164-15-706

**Published:** 2014-08-23

**Authors:** Shou-Guo Shi, Mei Yang, Min Zhang, Ping Wang, Yong-Xiang Kang, Jian-Jun Liu

**Affiliations:** College of Forestry, Northwest A&F University, Yangling, Shaanxi 712100 China; Life Sciences Department, Yuncheng University, Yuncheng, Shanxi 044000 China

**Keywords:** Transcriptome, Flavonoid biosynthesis, *Magnolia sprengeri*, Flower color

## Abstract

**Background:**

*Magnolia sprengeri* Pamp is one of the most highly valuable medicinal and ornamental plants of the Magnolia Family. The natural color of *M. sprengeri* is variable. The complete genome sequence of *M. sprengeri* is not available; therefore we sequenced the transcriptome of white and red petals of *M. sprengeri* using Illumina technology. We focused on the identity of structural and regulatory genes encoding the enzymes involved in the determination of flower color.

**Results:**

We sequenced and annotated a reference transcriptome for *M. sprengeri*, and aimed to capture the transcriptional determinanats of flower color. We sequenced a normalized cDNA library of white and red petals using Illumina technology. The resulting reads were assembled into 77,048 unique sequences, of which 28,243 could be annotated by Gene Ontology (GO) analysis, while 48,805 transcripts lacked GO annotation. The main enzymes involved in the flavonoid biosynthesis, such as phenylalanine ammonia-Lyase, cinnamat-4-Hydroxylase, dihydroflavonol-4-reductase, flavanone 3-hydroxylase, flavonoid-3′-hydroxylase, flavonol synthase, chalcone synthase and anthocyanidin synthase, were identified in the transcriptome. A total of 270 transcription factors were sorted into three families, including MYB, bHLH and WD40 types. Among these transcription factors, eight showed 4-fold or greater changes in transcript abundance in red petals compared with white petals. High-performance liquid chromatography analysis of anthocyanin compositions showed that the main anthocyanin in the petals of *M. sprengeri* is cyanidin-3-O-glucoside chloride and its content in red petals was 26-fold higher than that in white petals.

**Conclusion:**

This study presents the first next-generation sequencing effort and transcriptome analysis of a non-model plant from the Family *Magnoliaceae*. Genes encoding key enzymes were identified and the metabolic pathways involved in biosynthesis and catabolism of *M. sprengeri* flavonoids were reconstructed. Identification of these genes and pathways adds to the current knowledge of the molecular biology and biochemistry of their production in plant. Such insights into the mechanisms supporting metabolic processes could be used to genetically to enhance flower color among members of the *Magnoliaceae*.

**Electronic supplementary material:**

The online version of this article (doi:10.1186/1471-2164-15-706) contains supplementary material, which is available to authorized users.

## Background

**Figure 1:**
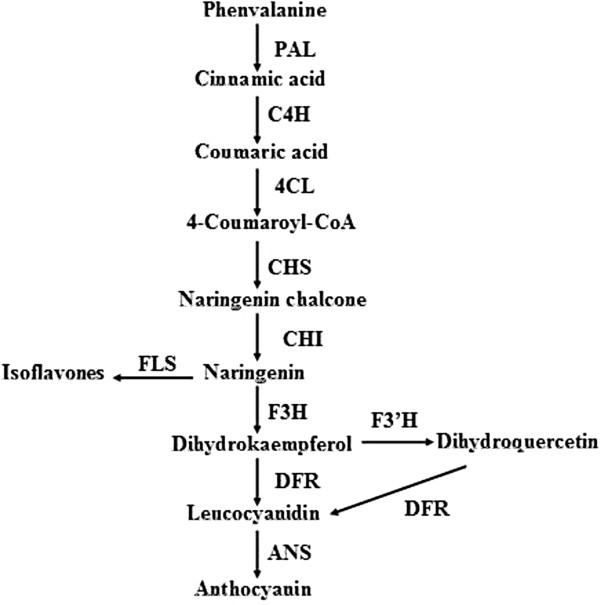
**Simplified scheme of the flavonoid pathway, comprising the general phenylpropanoid pathway, the anthocyanin branch, and other subgroups of flavonoid endproducts.** Abbreviations: PAL, phenylalanine ammonia lyase; C4H, cinnamic acid 4-hydroxylase; 4CL, 4 coumarate CoA ligase; CHS, chalcone synthase; CHI, chalcone isomerase; F3H, flavanone 3-hydroxylase; F3′H, flavanone 3′-hydroxylase; DFR, dihydroflavonol reductase; FLS, flavonol synthase; ANS, anthocyanidin synthase.

*Magnolia sprengeri* Pamp is one of the most valuable medicinal and ornamental plants of the Magnolia Family, which is native to the Qinling Mountains of Shaanxi Province and the Daba Mountains of Hubei Province, China [1]. The flower color (i.e., color of the petals) of *M. sprengeri* varies widely from white to red. Flower color has evolved via interaction with evolving pollinating insects. The extreme color variation ranges from pure white color, with only the faintest pale purple stripe on the base of the petals abaxially, to an intense red color on both sides. Previous studies showed that red and white were the two main types of abaxial color patterns. Anthocyanin is the primary flower pigment in higher plants, and its accumulation is tightly linked with flower development and color changes in most cases [2]. Natural phenotypic variations offer an opportunity to elucidate the role of anthocyanin genes that lead to extreme colors of *M. sprengeri.* Anthocyanin biosynthesis via the flavonoid metabolism pathway has also been well studied in flower development because of its high anti-oxidation properties and ultraviolet protection [3–7]. Anthocyanin is synthesized from phenylalanine, and catalyzed by phenylalanine ammonia-lyase (PAL), which is controlled by two groups of genes. The first group consists of the structural genes, including PAL, chalcone synthase (CHS), flavanone 3-hydroxylase (F3H), dihydroflavonol-4-reductase (DFR), anthocyanidin synthase (ANS), and UDP-glucose flavonoid 3-O-glucosyltransferase (UFGT), which represent the enzymes responsible for the biochemical reactions of anthocyanin synthesis [8–13] (Figure 1). The second group involves regulatory genes or transcription factors (TFs), which regulate the structural genes expressions during anthocyanin biosynthesis. The important TFs for anthocyanin biosynthesis belong to the MYB, bHLH, and WD40 families [14–21]. The coordinated expressions of the two categories of genes lead to anthocyanin accumulation during the color development process.

Transcriptome analysis of an organism is a particularly effective method for gene discovery, especially in non-model plants for which no reference genome sequences are available [22]. At the same time, it may provide powerful tools to identify differentially expressed genes, and its possible use in modern plant breeding continues to attract the attention of many plant biologists [23–26]. Sequencing technologies have dramatically accelerated genome-wide studies of transcriptomes and have been widely used to explore gene structure and gene expression, even in plants without a genome reference [27–29]. Illumina sequencing technology has been applied recently to transcriptome analyses of plant and animals, and can generate large amounts of sequence data cheaply and quickly [30–33].

**Figure 2:**
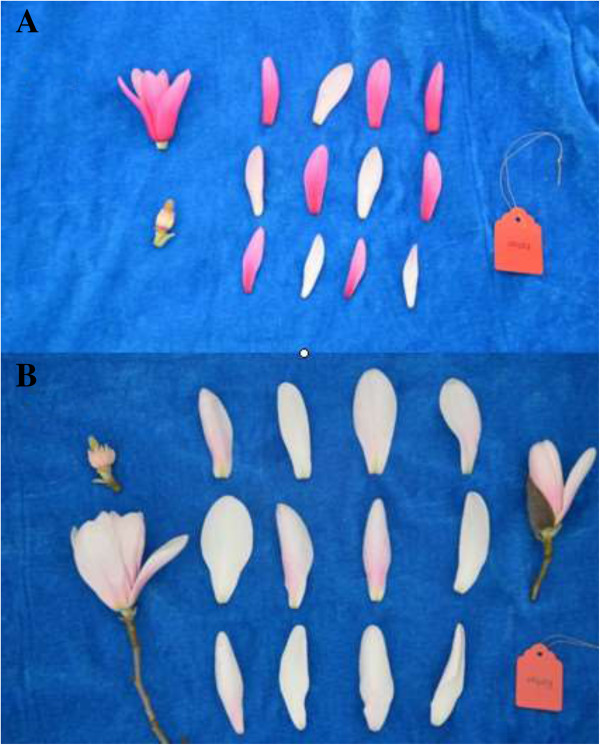
**White and red petals of** ***M. sprengeri.*** (**A**, Red petals; **B**, White petals).

In this study, we first sequenced the transcriptomes of white and red petals of *M. sprengeri* using Illumina technology (Figure 2). We focused on the discovery of structural genes and regulatory genes encoding enzymes involved in the anthocyanin biosynthetic pathway. We obtained sets of upregulated and downregulated genes from red and white flowers, and identified some candidate genes related to anthocyanin synthesis in *M. sprengeri*. The assembled annotated transcriptome sequences provide a valuable genomic resource to further understand the molecular basis of variations of flower color in *M. sprengeri.*

## Results and discussion

### Sequencing and sequence assembly

**Figure 3:**
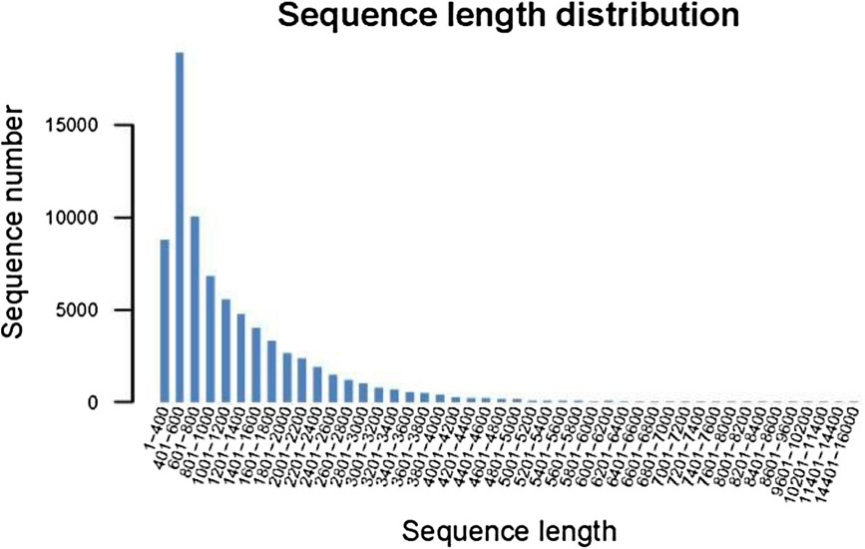
**Length distribution of sequencing reads and contigs of** ***M. sprengeri*** **.**

A cDNA library from red and white petals was sequenced using Illumina sequencing in a single run which generated 39,652,898 sequences with 4,004,9422,698 nucleotides (bp) from red petals and 68,698,774 sequences with 6,938,576,174 bp from white petals. After the removal of the low-quality raw reads (too short, empty, too many Ns), we obtained 39,652,898 (red) and 68,698,774 (white) high-quality sequences (Table 1). These short reads were assembled into 39,990 total genes, 77,048 total isogenes, and 91,433,742 total resides with an average length of 1,186.71 bp. The largest isogene was 15,888 bp and the smallest was 351 bp (Table 2). The sequence length distribution is shown in Figure 3.

**Table 1:** **Summary of sequencing for** ***M. sprengeri***

Sequencing	No. of sequences	No. of bases	No. of high-quality reads	No. of bases
Red petal	39,652,898	4,004,942,698	35,642,032	3,026,511,514
White petal	68,698,774	6,938,576,174	62,964,028	5,246,524,133

**Table 2:** **Splicing results for** ***M. sprengeri***

Type	Sum
Total genes (n)	39,990
Total isogenes (n)	77,048
Total residues (bp)	91,433,742
Average length (bp)	1,186.71
Largest isogene (bp)	15,888
Smallest isogene (bp)	351

In this study, we obtained 35,642,032 sequences and 62,964,028 high-quality sequences in red and white petals of *M. sprengeri*, respectively (Table 2). By comparison, the assembly of 39,990 *M. sprengeri* sequences from GenBank (using the GS De novo Assembler) led to only 77,048 unique sequences. The unique sequences derived from GenBank sequences and Illumina sequences were compared by a BLAST search, where matches were defined as having an identity > 90% and an overlap >100 bp. Our Illumina sequencing efforts produced 77,048 unique sequences. Unique sequences that were not present in GenBank were considered as the novel transcripts of *M. sprengeri*. The large quantity of unique sequences should cover the vast majority of genes from *M. sprengeri* petals, providing, for the first time, a powerful gene resource for this medicinal and ornamental plant.

### Gene ontology (GO) annotation

**Figure 4:**
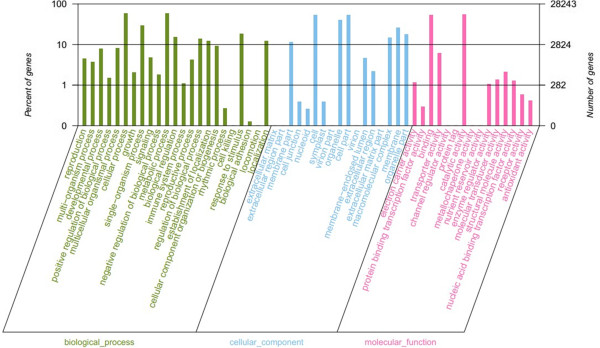
**Histogram representing Gene Ontology (GO) classification.** GO categories, shown on the x-axis, were grouped into three main ontologies: biological process, cellular component, and molecular function. The right y-axis indicates the number of genes in each category, while the left y-axis indicates the percentage of total genes in that category. The ‘all gene’ indicates that the unigenes were those assembled from reads from the red and white sample.

GO annotation provides a description of gene products in terms of their associated molecular functions, cellular components, and biological processes [34]. GO functional interpretations for plants are primarily based on the *Arabidopsis thaliana* genome. GO terms were assigned to 28,243 *M. sprengeri* sequences based on sequence similarities with known proteins and annotated using The Arabidopsis Information Resource (TAIR) using GO slim. Among the 77,048 spliced transcripts, 28,243 had GO annotated transcripts while 48,805 had no annotated transcripts (Figure 4). The GO annotations of the unique sequences were most frequently related to molecular function (23,227 unique sequences), followed by biological processes (20,540 unique sequences) and cellular components (16,690 unique sequences). For each sequence, the specifically annotated GO terms provide a broad overview of the groups of genes cataloged in the transcriptome. Finally, the functions of the identified transcripts were determined to be involved different biological processes. The best represented groups were protein metabolism, developmental processes, response to abiotic or biotic stimuli, and response to stress and transport. These GO annotations provided valuable clues to investigate the specific processes, molecular functions, and cellular structures of the *M. sprengeri* transcriptome.

### Clusters of orthologous group (COG) and eukaryote clusters of orthologous groups (KOG) classification

**Figure 5:**
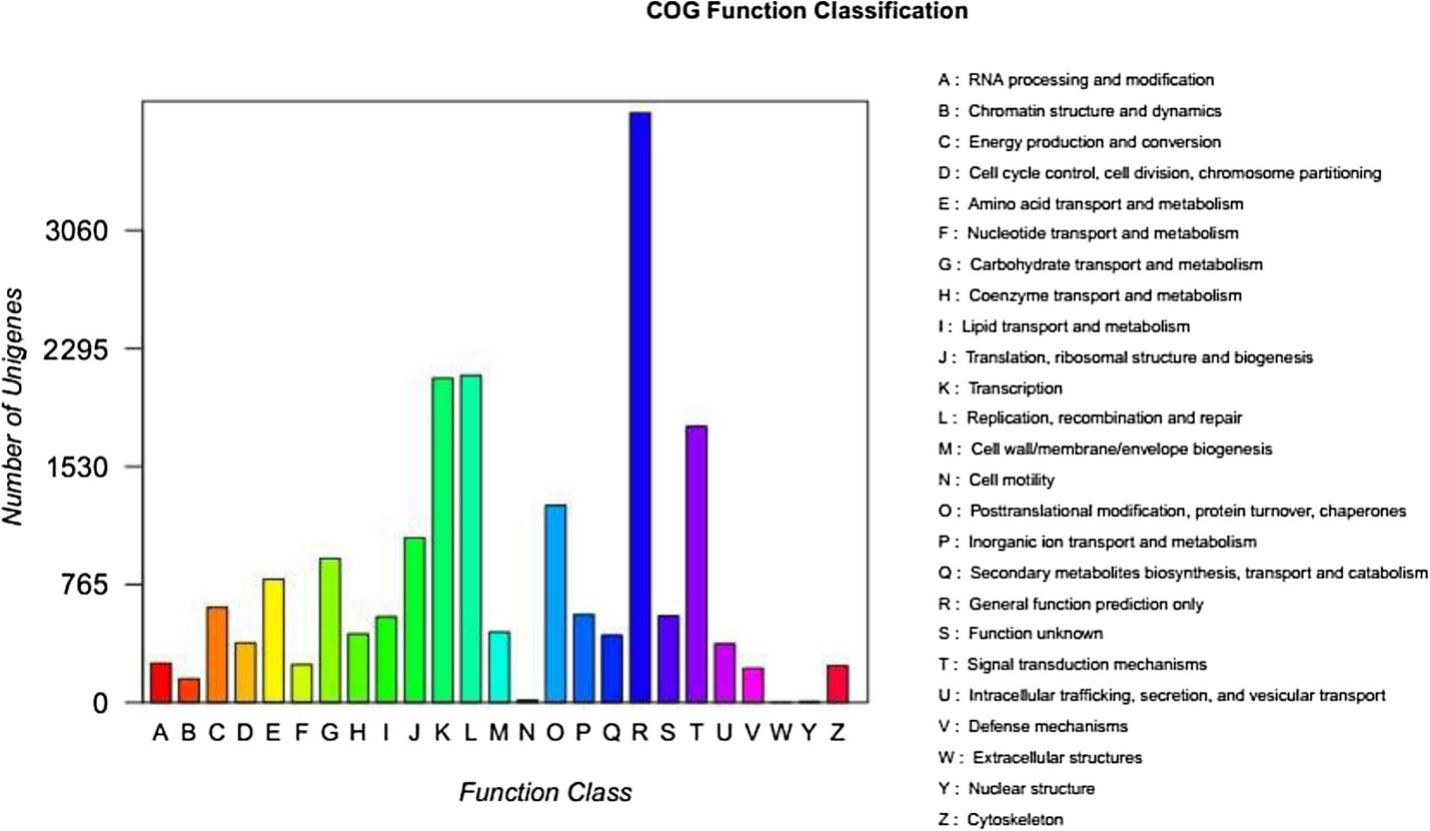
**Histogram representing clusters of orthologous groups (COG) classification.** A total of 25,626 unigenes were assigned to 24 categories in the COG classification. The y-axis ‘Frequency’ indicates the number of genes in a specific functional cluster. The right side legend shows a description of the 24 functional categories.

**Figure 6:**
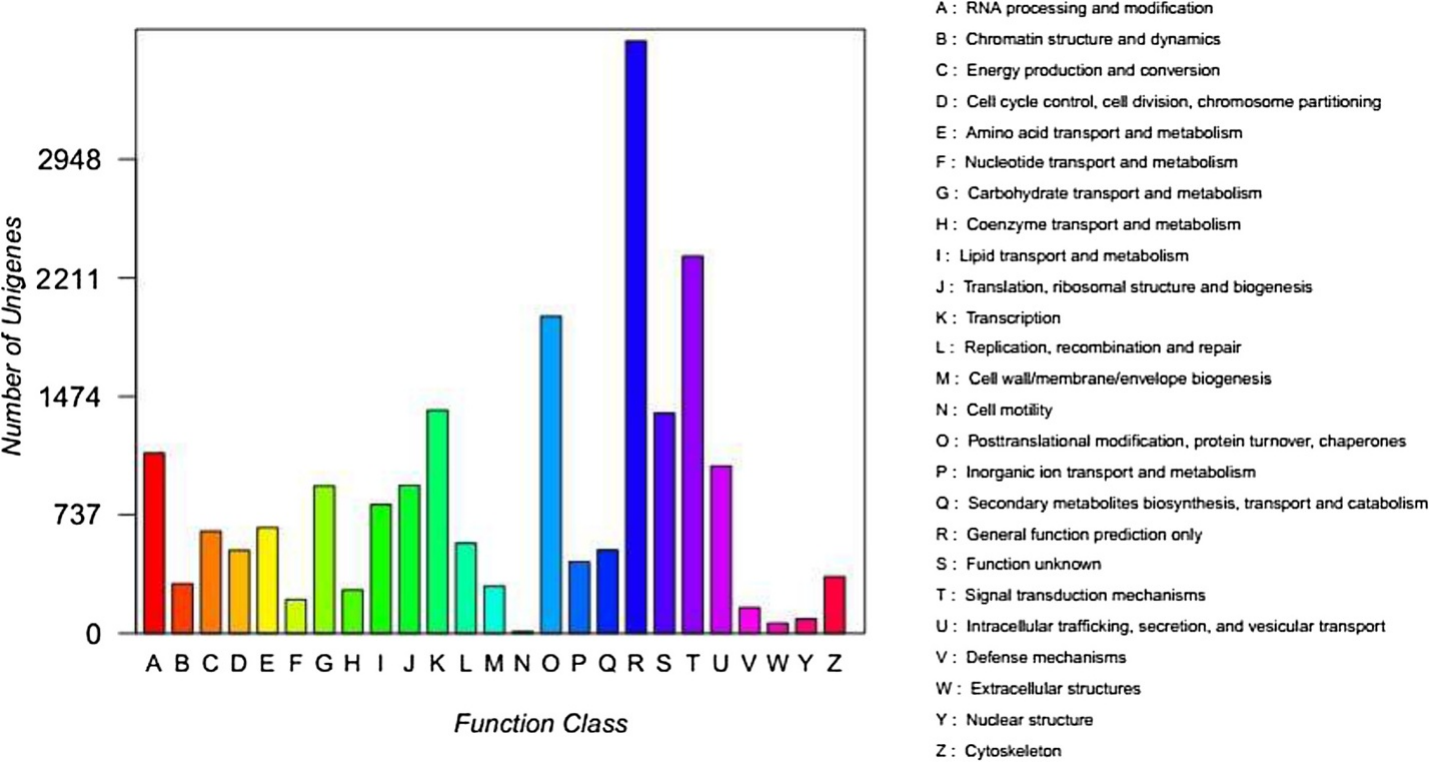
**Histogram representing clusters of orthologous groups (KOG) classification.** 25,626 unigenes were assigned to 25 categories by KOG classification. The y-axis ‘Frequency’ indicates the number of genes in a specific function cluster. The legend on the right shows a description of the 25 functional categories.

The GO analysis identified well-represented categories within the cellular component group, including sequences related to the chloroplast, the plasma membrane, and the ribosome. Additionally, the sequences encoded a broad set of transcripts that could be assigned to molecular function categories. To further examine the integrity of our transcriptome library and the effectiveness of the annotation process, we identified the unigene numbers with COG and KOG classification. Altogether, there were 25,626 unigenes identified from all unigenes with COG (Figure 5) and KOG classifications (Figure 6). Among the 24 COG categories, the cluster of “General function prediction” accounted for the largest proportion (3,823, 14.9%) followed by “Replication, recombination and repair” (2,120, 8.3%), “Transcription” (2,102, 8.2%) and “Signal transduction mechanisms” (1,792, 7.0%). The categories of “Nuclear structure” (7) accounted for 0.03%, “Secondary metabolites biosynthesis, transport and catabolism” (437) accounted for 1.7%, and “RNA processing and modification” (254) accounted for 1.0%. Among the 25 KOG categories, the cluster of “General function prediction” accounted for the largest proportion (3,682, 14.4%), followed by “Signal transduction mechanisms” (2,346, 9.2%), “Posttranslational modification, protein turnover, chaperones” (1,971, 7.7%) and “Transcription” (1,388, 5.4%). The categories of “Nuclear structure” (90, 0.35%), “Secondary metabolites biosynthesis, transport and catabolism”(519, 2.0%), “RNA processing and modification” (1,121,4.4%), and “Extracellular structures” (62, 0.24%) had the least proportions.

### Pathway assignment based on the Kyoto Encyclopedia of Genes and Genomes (KEGG) classification system

The KEGG classification system provides an alternative functional annotation of genes according to their associated biochemical pathways [35]. KEGG annotations for *M. sprengeri* transcripts were based on sequence similarity searches against the KEGG database, and matches were assigned the corresponding enzyme commission (EC) number. Overall, 12,082 *M. sprengeri* unique sequences were assigned KEGG annotations, of which only 1,696 unique sequences were assigned to the biosynthesis of secondary metabolites pathways.

**Figure 7:**
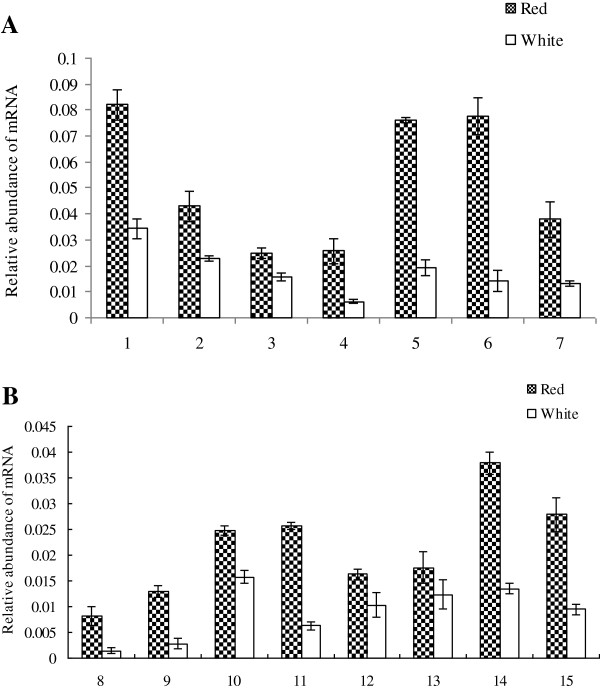
**Changes in transcript levels of genes of red and white petals. A**. Relative transcript levels of regulatory genes in red and white petals. 1: Gene 55739; 2: Gene 53876; 3: Gene 43573; 4: Gene 47103; 5: Gene 49650; 6: Gene 50438; 7: Gene 51086; 8: Gene 25552. **B**. Relative transcript levels of transcript factors in red and white petals. 9: Gene 45264; 10: Gene 45298; 11: Gene 46821; 12: Gene 47225–0; 13: Gene 47225–1; 14: Gene 47747; and 15: Gene 50161. The amount of transcript was normalized to the level of *M. sprengeri act* gene. Mean values and standard errors (bars) were obtained from three independent experiments per timepoint.

Metabolic pathways were well represented among *M. sprengeri* unique sequences, most of which were associated with amino acid metabolism, galactose metabolism, biosynthesis of secondary metabolites, aminoacyl-tRNA biosynthesis, and flavonoid biosynthesis. Notably, the transcripts encoding all the enzymes involved in the flavonoid biosynthesis pathway were present in our Illumina sequences dataset (Table 3). The expression of all candidate genes in the petals (Additional file 1: Figure S2) was confirmed by quantitative real-time PCR (qPCR) (Figure 7).

**Table 3:** **Genes encoding enzymes involved in flavonoid biosynthesis in** ***M. sprengeri***

Name	Description	Number of transcripts KO no.	EC no.
*PAL*	PhenylalanineAmmonia-Lyase	K10775	EC.4.3.1.5
*C4H*	Cinnamat-4-Hydroxylase	K13065	EC.1.14.13.11
*DFR*	Dihydroflavonol-4-Reductase	K13082	EC.1.1.1.219
*F3H*	Flavanone 3-Hydroxylase	K00475	EC.1.14.11.9
*F3′H*	Flavonoid-3′-Hydroxylase	K05280	EC.1.14.13.21
*CHI*	Chalcone Isomerase	K01859	EC.5.5.1.6
*FLS*	Flavonol Synthase	K05278	EC.1.14.11.23
*CHS*	Chalcone Synthase	K00660	EC.2.3.1.74
*ANS*	Anthocyanidin Synthase	K05277	EC.1.14.11.19

### Candidate genes encoding enzymes involved in the biosynthesis of flavonoids

Flavonoid biosynthesis is an integral part of secondary metabolism; therefore, it should be considered within the context of cellular metabolism. The color of the petal is affected by the flavonoids metabolism pathways. Changes in the transcript abundances of the genes encoding enzymes in these pathways are listed in Tables 3 and 4.

**Table 4:** **Differentially expressed genes related to flavonoid biosynthesis in** ***M. sprengeri*** **red and white**

Transcript	Annotation	FPKM-R	FPKM-W
comp55739_c0_seq2	Phenylalanine Ammonia-Lyase	967.65	454.39
comp53876_c0_seq1	Cinnamat-4-Hydroxylase	447.91	143.63
comp43573_c0_seq2	Dihydroflavonol-4-Reductase	264.97	7.46
comp47103_c0_seq1	Flavanone-3-Hydroxylase	420.34	389.55
comp49650_c0_seq1	Flavonol synthase	786.51	299.47
comp50438_c0_seq1	Chalcone synthase	705.94	225.14
comp51086_c0_seq1	Anthocyanidin reductase	344.97	203.23

FPKM-R, fragments per kilobase of transcript per million fragments mapped red; FPKM-W, fragments per kilobase of transcript per million fragments mapped white.

The levels of transcripts encoding the first enzymes in the flavonoids biosynthesis, such as PAL (EC.4.3.1.5), were markedly higher in red petals than in white petals. The transcript abundance of the flavonoids biosynthesis enzymes, including C4H (EC.1.14.13.11), DFR (EC.1.1.1.219), F3H (EC.1.14.11.9), flavonoid-3′-hydroxylase (F3′H), flavonol synthase (FLS, EC.1.14.11.23), CHS (EC.2.3.1.74), and ANS (EC.1.14.11.19), were also higher in red petals.

The Plant Transcription Factor Database was used to search the *M. sprengeri* transcripts dataset to identify the genes encoding putative TFs or transcriptional regulators [36]. A total of 270 transcripts were predicted to be TFs and were sorted into three families (data not shown). Of these genes, the expression of eight *MYB* genes showed 4-fold or greater changes in red petals compared with white petals. Further studies are needed to determine whether the changes in transcript abundance of these putative TFs could be related to the regulation of flavonoid metabolism.

Flavonoids are a large group of polyphenolic compounds and are a structurally diverse class of plant secondary metabolites. They are important for defense against pathogens and herbivores, protection from harmful ultraviolet radiation, and flower pigmentation for attracting pollinators [37–39]. In addition to their physiological functions in plants, flavonoids display a wide range of anti-oxidant, anti-microbial, anti-inflammatory, and anti-cancer activities [39]. As a dietary component, flavonoids are considered to have health-promoting and disease-preventing properties. Recently, flavonoids have been intensively investigated as potent pharmaceuticals for treating chronic human pathological conditions [40–44].

According to the present transcriptomic analysis, the expression of eight genes was altered in flavonoid biosynthesis, including PAL, C4H, F3H, FLS, CHS, DFR, and ANS. It has been known for decades that flavonoids are synthesized from phenylalanine. This process involves three steps: first, PAL, cinnamic acid 4-hydroxylase, and 4-coumarate coenzyme A ligase catalyze the conversion of phenylalanine to; second, CHS, CHI, F3H, F3′H, and DFR catalyze the conversion of *p*-coumaroylCoA to leucoanthocyanidins; and third, ANS catalyzes the conversion of leucoanthocyanidins to form anthocyanidins [9]. In this process, any changes in the expression of the genes encoding these enzymes can lead to the production of different anthocyanidin species [45–47], and such changes may lead to the production of the red petal phenotype of *M. sprengeri*. Our study also identified certain genes encoding TFs related to flavonoid biosynthesis, such as MYBs, bHLHs, and WD40s. The differentially expressed structural genes and differentially expressed TF genes that may be associated with flavonoid biosynthesis are shown in Tables 4 and 5. Such information would help provide a deeper understanding of how changes in gene expression are related to the changes in the color of *M. sprengeri* flowers.

**Table 5:** **Changes in transcript abundance of predicted transcription factors and regulators about flavonoid biosynthesis in** ***M. sprengeri*** **red and white**

Transcript	Annotation	FPKM-R	FPKM-W
comp25552_c0_seq1	MYB domain protein 20	2.54	0.7
comp45264_c0_seq2	R2R3 MYB transcription factor	13.27	0.35
comp45298_c0_seq2	R2R3-MYB transcription factor MYB9	92.49	1.02
comp46821_c0_seq4	MYB6	39.37	9.38
comp47225_c0_seq1	MYB-related protein 306	38.92	7.95
comp47225_c1_seq1	MYB-related protein 306 isoform 1	42.62	13.5
comp47747_c0_seq2	MYB transcription factor	89.68	17.14
comp50161_c1_seq1	R2R3 Myb24 transcription factor	288.96	72.35

FPKM-R, fragments per kilobase of transcript per million fragments mapped red; FPKM-W, fragments per kilobase of transcript per million fragments mapped white.

Based on this comparison, almost all of the candidate genes involved in the flavonoid biosynthesis were present in the transcriptome datasets of *M. sprengeri* in this study. These results highlight the immense capacity of high-throughput sequencing to discover genes in metabolic pathways.

### Anthocyanin accumulation

**Figure 8:**
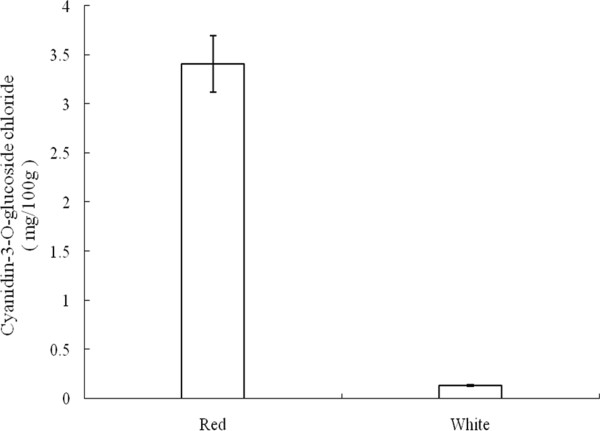
**Accumulation of anthocyanidin in petals of red and white** ***M. sprengeri*** **.** Red: Anthocyanidin concentrations in the petals of the red flower; White: Anthocyanidin concentrations in the petals of the white flower. Concentrations in the petal of the red and white flowers were determined by measuring the absorbance at 350 nm using HPLC analysis with cyaniding-3-O-glucoside chloride as the standard. Data are the mean ± SD of three replicates.

Anthocyanin accumulation is tightly linked with flower development and color changes in most cases [2]. To examine the accumulation of anthocyanin in the petals of red and white *M. sprengeri*, the flower extracts were subjected to high-performance liquid chromatography (HPLC) analysis. The HPLC data showed that the main anthocyanin in the petals of *M. sprengeri* is cyanidin-3-O-glucoside chloride. The accumulation of cyanidin-3-O-glucoside chloride in petals of red and white M. sprengeri was 3.421 and 0.132 mg per 100 g samples, respectively (Additional file 2: Figure S1), Our results indicate that red petals accumulate 26-fold more cyanidin-3-O-glucoside chloride than white petals (Figure 8).

## Conclusions

Illumina next-generation sequencing technology was used for sequencing and transcriptome analysis of the non-model plant *M. sprengeri* pamp. We identified the genes encoding key enzymes and reconstructed the metabolic pathways involved in biosynthesis and catabolism of flavonoid of *M. sprengeri.* Our results promote understanding of the mechanisms underlying various metabolic processes, and will enable the genetic manipulation of flower color in *M. sprengeri*.

The accumulation of flavonoids and the discovery of genes associated with their biosynthesis and metabolism in *M. sprengeri* are intriguing and worthy of further investigation. The sequences and pathways identified here represent the genetic framework required for further studies. Quantitative transcriptomics in concert with physiological and biochemical analysis in *M. sprengeri* under conditions that stimulate production and accumulation of flavonoids could help provide insights into the regulation of, and links between, these pathways.

## Methods

### Plant materials

The petals of red and white *M. sprengeri* were harvested from approximately 50-year-old trees in March 2012 from Wufeng County, Hubei Province, China (Figure 2). We selected 10 trees with red flowers and 10 trees with white flowers for petal collection. Nine petals of each color were selected for RNA-sequencing experiments while three petals (around 0.5 g) of each color were taken for the HPLC experiments. For qPCR, we used additional 5–10 petals to isolate total RNA. After cleaning, the petals were cut into small pieces, immediately frozen in liquid nitrogen, and stored at -80°C until further processing.

### RNA preparation

The TRIzol® reagent (Invitrogen) was used to extract total RNA from the petals of red and white *M. sprengeri* according to the manufacturer^’^s instructions (Invitrogen, USA). The purity of all RNA samples was assessed at an absorbance ratio of OD260/280 and the RNA quality was tested using a 1% ethidium bromide-stained (EtBr-stained) agarose gel. A GeneQuant100 spectrophotometer (GE Healthcare, UK) assessed the RNA concentration before processing.

### cDNA synthesis and Illumina sequencing

Clontech’s SMART cDNA synthesis kit (Clontech, USA), was used to produce first-strand cDNA from 5 μg of total RNA extracted from the petals of *M. sprengeri*, according to the manufacturer’s instructions. The samples were treated with RNase-free DNase I (Takara Biotechnology, China). To construct a cDNA library, oligo (dT) magnetic beads were used to purify poly (A) mRNA from total RNA. The RNA was then fragmented into small pieces by the addition of fragmentation buffer. These short fragments served as templates to synthesize first-strand cDNA using random hexamer primers. Second-strand cDNA was synthesized using buffer, dNTPs, RNaseH, and DNA polymerase I. A QiaQuick PCR extraction kit purified the short fragments. These fragments were washed with elution buffer for end repair and poly (A) addition and were then ligated to sequencing adapters. Suitable fragments, as judged by agarose gel electrophoresis, were selected for use as templates for PCR amplification. An Illumina HiSeq™2000 sequenced the cDNA library using paired-end technology in a single run.

### Transcriptome assembly and annotation

The Solexa GA pipeline 1.6 generated the transcriptome de novo assembly. After the removal of low-quality reads, the Trinity de novo assembler (http://trinityrnaseq.sourceforge.net/) [48, 49] assembled processed reads with an identity value of 95% and a coverage length of 100 bp [48, 49]. First, the overlap information in the short reads was used to construct high-coverage contigs, and then the short reads were assembled into contigs. We then realigned the short reads onto the contigs and estimated the distance and relation of the two contigs using the pair-end linkage and insert size information. Unreliable linkages between the two contigs were filtered and the remaining contigs with compatible connections were linked to each other, and had at least three read-pairs. The last step was to close gaps in the scaffolds. We gathered the paired-end reads with one end mapped to the contigs and another end located in the gaps and performed local assembly with the unmapped end to extend the contig sequence into the small gaps in the scaffolds. CAP3 [50] was used (with default parameters) to reduce redundancy and to combine scaffolds and single-end contigs in the separate assemblies.

To annotate the *M. sprengeri* transcriptome, we performed a BLAST search against the non-redundant (NR) database in NCBI, SWISS-PROT, KEGG, and COG with a cut-off E-value of ≤10^-5^. The Blast2GO software (http://www.blast2go.com/b2ghome) obtained the GO annotations and the corresponding EC numbers of the sequences.

### Pathway assignment with KEGG

Pathway assignments were mapped according to the KEGG database (http://www.genome.ad.jp/kegg/kegg2.html) (versionKEGG) [51]. EC numbers were assigned to unique sequences that had BLASTX scores with an E value cut-off of 10^-5^ after searching the KEGG protein databases. The unique sequences were mapped to specific biochemical pathways according to the corresponding EC distribution in the KEGG database.

### Quantitative Real-time PCR (qPCR) Analyses

To remove any contaminating genomic DNA before cDNA synthesis, we treated the total RNA with RNase-free DNAse I (Invitrogen, USA) according to the manufacturer’s instructions. A NanoDrop™ 1000 spectrophotometer was used to quantify the RNA before and after this DNAse I treatment, and RNA quality and integrity were checked by electrophoresis using agarose gels stained with ethidium bromide. For qPCR, first-strand cDNA was synthesized with 2 μg of total RNA in a volume of 20 μL, using a SYBR®PrimeScript™ RT-PCR Kit II (Takara*,* China) plus random hexamers and oligo(dT) primers. After reverse-transcription, the reaction product was diluted 10-fold with sterile water. Real-time PCR was performed on an iQ5 instrument (Bio-Rad, USA) using SYBR Green qPCR kits (Takara*,* China) according to the manufacturer’s instructions. Primer sequences are listed in Table 6. Real-time PCR reactions were carried out in 20-μL volumes containing a 10-μM concentration of each primer, 40 ng of cDNA, and 10 μL of SYBR Premix Ex Taq™ II. Thermal-cycling conditions included an initial heat-denaturing step at 95°C for 3 min; then 40 cycles of 95°C for 20 s, 58°C for 20 s, and 72°C for 20 s. Fluorescence was measured at the end of each cycle. A melting-curve analysis was performed by heating the PCR product from 58°C to 95°C. Expression data were presented as relative units after normalization to the *act* control, using the 2^-△△CT^ method. Values for mean expression and SD were calculated from the results of three independent experiments.

**Table 6:** **Primers used in quantitative real-time PCR**

Gene ID	Primer sequence(forward) 5′-3′	Primer sequence(reverse) 5′-3′
55739	TAACGAAGCCGAAACAGGA	GAGAATTGGGCGAACATCA
53876	ACGCATCTTACGCCAGTG	ATTCCAGCCGTTCATTCT
43573	TCGTGGAAGCGTGCGAGGAC	AGCGTGATGGTGCCAGGGTC
47103	CGGTTCGTGGCTGGTTAT	TCCGCTAGTGATTTGGAGAC
49650	GCAGAAACAATCCATCCCTCAA	CAGGACGACAGTAAACAAGGAGAA
50438	GCAGGCATCCAAGCAATAC	AATAATCCTCCCACTCAAGC
51086	GGGGACTCTACACCAGGAA	CTAACGGAGGAGATATTGACG
25552	GCCTCACATAACCTTTCTC	TTGACCCTTTCAGCCAGTA
45264	CAATCGGTGTCGTAAGAGC	CCCGTCGTAATGGAAAGTA
45298	TTTATTTAGTGCCGATACCA	ATTACGATGTGCCAGGAG
46821	GCGAATCATACTCCGACAT	TTGCTGCTTTGACTCTGC
47225	ATGCGTAGGTAGATGGTTG	CACTGATACTGAGGAGGAGAA
47747	CCGAAGAAGATGCGACAAA	GAGCAGCGATTCAAGAGCC
50161	GGCTTGATTTGGGAGACGA	TACCGACCTGTGGCGAGAA
53953(Actin)	GGCTGGATTTGCTGGAGAC	GTGGTGCTTCGGTGAGGAG

### HPLC analysis of anthocyanin

*Magnolia sprengeri* petals (0.5 g) were ground in 1.5 mL of 70% methanol containing 2% formic acid at 4°C, then centrifuged at 10,000 g for 10 min at 4°C. The supernatant was passed through a 0.22-μm syringe filter before HPLC analysis. Anthocyanins were investigated on an Agilent 1100 HPLC equipped with a diode array detector (Agilent Technology), as described by Zhang et al. [52]. The total anthocyanin concentration was calculated based on a cyanidin-3-O-glucoside standard (Sigma-Aldrich, St. Louis, MO, USA).

## Electronic supplementary material

Additional file 1: **Full-length cDNA sequences of genes used in qPCR assay.** (DOC 59 KB)

Additional file 2: **Anthocyanidin analysis of petals in red and white flower color.** Peaks of HPLC were identified by retention time compared with cyaniding-3-O-glucoside chloride standards. (A: Red petals; B: White petals). (DOC 124 KB)
