# Supplementary material for: Genome-wide transcriptome analysis of genes involved in flavonoid biosynthesis between red and white strains of Magnolia sprengeri pamp
Source: BMC Genomics. 2014 Aug 23;15(1):706. doi: 10.1186/1471-2164-15-706 (PMC4156625; doi:10.1186/1471-2164-15-706)
Supplement: Supplementary file 1 — Additional file 1: Full-length cDNA sequences of genes used in qPCR assay. (DOC 59 KB) [file 12864_2014_6403_MOESM1_ESM.doc]

1. comp55739_c0_seq2

GACTCGACTCGAAAACTCGCACTCGGTACTCAACTCGTGTGCTCGTACTCGAACTCGCCT

CGAAAACTCGGACTCGACTCGACTCGTTTACTACTCGTACTCGGCTCGTACTCGAGCGAG

TAGAGTACGAGTAGGGAATCCATGCTCGTTGGCTGAGTAGAGCCGAGTACGAGCAACACT

CGGGCGTTAAGAGTCGAGTACGAGTAGGATGATACTCAACTCGACTCGACTCGTGTACAA

CTCTAGTTGCAACTGTGACCCGGTTCCAAAAATATGCTCATCAGATCATCATCATCATCC

AATCAATGTACTTTTTCCCCTCAAATAGAAACTACTGGGCCATTTTGTTCCGTCCATCCT

TTTACAGGTCAAAGATCAGCCAATCAGGATGATCAACGGTTGAAATTTTGTTCTATCATT

TATTGACAATAAGACCCAATTGACGAACCGTCTCGATCACAATGCACGGACCCCACTCAA

TAATTGAAATAAGGGGACTACCAATTTAGATGGTACCGCAGGCTACGATATTTCTCCACC

CACACATAACACATGCCAAACCCGAGTATCTTTTCTACCCAGTCAATGGTCCAAAATAAA

AAAAGGCCCACCAAACACCTTTTCATTACAAACAGCCAATTGAAAGTTTCCACTCTCCTT

CCATGCAAGTGGTCACAAAAGGCACCCAAGTCCACCAACCCCATTTGGATTGACTAGACC

ATAGACCGCAGCCTGGGACCATTGGGTCCCATTCCCCCCATCAACCTCTATAAAATCCAC

CCCACTCCTATCATTCCACTCACCCATTCATTTGATATCCTTCTCATAATCTCCCTCTCA

TCTCCTCCTTTAAAATTCCAAACCCCACCTCAACCAATGGCCCACAAGAACAACAGCATC

CACCGTCCAATCGACTCCTGCCGTCCTCCAACACTCCCCCTCCCAAAAGACGGCAAGAAC

CCAATCTCCGTCTCCGGTACTTCCTACGTCTCAACTCCTCCCCACTGGAAAAAAGCAGCG

GAGGCCCTTCAGTGCACTCACTTCGATGAAGTCCGTCGCATGGTGGCCCAGTTCCAGCAA

TCCCAGACCGTTGATCTCCAAGGGACCACACTTACTGTAGCCCAAGTCGCCGCCATTGCT

CGTCGGCCCATCGTGACCGTCCAACTCGATGAGGCTGCCGCACGTGCTCGTGTAACAGAA

AGCGCCACGTGGGTGGCAGACAACATCGCTCGTGGTGCTGACGTGTATGGAGTCACTACC

GGATTCGGTGCAACCTCACAACGGCGTACTAACAAGACCGCCGATCTGCAAACGGAACTG

ATCCGATTCCTCAACGCTGGTGTGATCGGAAAGGAGATGCTACCAACGAGCTATTCAAAA

GCGGCGATGCTCGTTCGAACGAATACACTGATGCAGGGCTACTCCGGCATCCGATGGGAG

ATTCTCGATGCCATGGCCAACCTCATGAACAAAAACCTAATTCCCAAACTCCCAATGAGG

GGGACTATCACTGCATCAGGAGATCTCGTCCCTCTATCCTACATTGCTGGTGTTATCACT

GGCAGGCACAATTCTCGAGTCGTCACTCCAGAAGGTGAGGAGATTACAGCATTGGAAGCA

CTGAATCGAGCTGGGATTGCAGGCCCGTTCGAGCTACAAGCGAAAGAAGGGCTAGCGCTT

GTTAACGGAACTGCTGTGGGATCTGCTGTCGCAGCTACTGTCTGCTTCGATGCGAATGTT

TTGGTTCTGCTTTCTGAAATCCTATCGGCGATGTTTTGTGAAGTGATGCAAGGGAAGCCG

GAATTCACGGACCCTCTGACGCATGAGCTCAAACACCACCCGGGCCAGATCGAGTCGGCG

GCGATTATGGAATACCTGCTCGACGAGAGCGATTACGTTAAAGAGGCGAAGGCTCGTCAT

GAGACGAACCCGCTAACGAAGCCGAAACAGGATCGGTACGCGCTTCGGACTTCACCGCAA

TGGCTCGGACCACAGATTGAGGTGATCCGTGCAGCGACTCATTCGATCGAGCGGGAGATT

AACTCCGTGAACGATAATCCTCTGATCGACGTGGCCAGGGATATTGCCCTCCATGGCGGG

AACTTCCAAGGAACTCCGATTGGGGTTTCGATGGATAATCTCCGTATCGCTCTGGCTGCA

ATTGGGAAACTGATGTTCGCCCAATTCTCCGAGCTGGTCTGTGATTACTACAACAACGGA

TTGCCGTCGAATCTCAGCGGCGGACCTAACCCGAGCTTGGATTATGGATTCAAAGGGGCT

GAGATCGCGATGGCAGCTTACTGCTCAGAGCTTCAGTACCTGGCGAATCCGGTAACGACG

CACGTGCAAAGCGCTGAGCAGCATAACCAGGACGTGAATTCGCTGGGACTGATCTCAGCC

CGCAAGAGCGCTGAAGCTATTGAGATCCTGAAGCTGATGTCTTCAACGTACATGGTCGCG

CTCTGCCAAGCTGTCGATCTCCGACACCTTGAGGAGAACATGCGGGAGGTTGTGAAGCAT

GTCGTCCTGCAAGCGTCGAGGAAGACGCTCTACTCTGATAATGATGGGTCCCTGCTCGAG

TCTCGATTCTGCGAGAAGGAGCTGGTGCAGGTGATCGAAAATCAGCCCGTTTTCTCTTAC

CTCGACGACCCCACGAATCCGTCTTACTCTCTGATGCTCCAGCTTCGGGAAGTTCTCGTC

GAGAAATCTTTCGAAGAGCGGAAGTCAGGCGGCGAAGGTGCTGATGGAGAAGACGGTTAT

TCGGTTTTTAAACGGATACCTGTTTTTCAGAAAGAGGTGAAGGAGAGGCTGGAGGAGGAA

GTGCCGAAGGCGAGGGAGAGATTCGACGGTGGGGATTTTCCGATTGCGAATAGGATAAAG

AAGTGTAGGACTTACCCGATTTATCATTTTGTGAGGACTGAGGTGGGGACCACATTGCTG

AGTGGGGCCCGGAAGGTGAGTCCTGGTGAGGATATTGAGAAGGTTTATGATGGTATTGCT

GATGGGAAGCTTGGGGAGGTGATGATGAAGTGCTTGGCATTCTGGCGTGGGTCTGCTGGA

CCGTTCACGCCGCGGTCTGCTGTTGCATCGCCAGCTCAATGTAATCCTGAGTATTGGGGG

TGGTTTGATAACGTGAGGTCACCGTCTGCTACTAGTGGGAGAGGGTATTGGAACCTTTGA

ACGGAGTGGATCAGATGATGGATTGAAGGATGGACGGCTGGATGATTATGTTTTGCTTTT

GGGTTTCATTTTTATTTTTGAAAGGTGATTTTATGGTGATATTATCGTTGAATGTAAGTG

ACTTCATAAAAGAGAATATTTGAATGGAAGACTTTGTTTTTATGTGGAAAAAAAA

2. comp53876_c0_seq1

AATTTTCTCTCAACCATTCATTTACCTTATACACACATAGCCTACCAATAATCATTTCAT

TTGGGCCAATTAATAGAAACATGGATCAGCTGATGGCCGGCTCTGATCTCCCACCCATGT

GAGCAAGAGACCCCGAACCATACACGTTTTATTGCAACTTTGCAATGTGCCCATACAAAT

CCATACATTTACGCTCATCCACCATTCCATCCTCTTACCAACACCATCCACCATACAAAT

CCAAAGAAATAAGATAGAAAGAGTCCTTTAAAAAGTAAACATAGGATCCAAAAAGGAAAA

AAGAAGGAAATAAGAGCCTCAAAACACTCTAGGTTTTGCAATAATGGTGGAATGCTTGAG

TATGTGCAAGCTGAACTGCCCGCCTTTCTCACTTGTATCGAGCTTAGCCTGACCGACCGG

CGGCATGAGCTCGAAATTCTGCACCAGGCGGCCCAGCGTGATCCCGAGAATCGGCAATGC

CAGTATGATCCCTGGGCAGCTCCTGCGGCCCACCCCGAATGGCAGGTAGCGGAAATCATT

GCCATTAGCTTCCACATTGGCCTCCTCCTCGAGGAACCGCTCGGGGCGGAACTCCTCGGG

CTTCTTCCATTGCTTGGGGTTGTTTGCTAAGAACCATGCATTGACTAGGATCTTGCTCTC

GGCCGGGATGTCGTAGCCTGCCAGCTTAGCGTCGTTCAAGTTCATGTGTGGGACAAGAAG

TGGGATGGCCATGCGGAGCCTGAGCGTCTCCTTGATCACTGCCTGGAGGTAGGGGAGCTT

GTAGGTGTCTGGCTCAGTGACCTGGACGCCGGGGCCTAGGACCGTGTCGAGCTCGTTACG

CAGCTTCTGTTGGATCTCGGGGTGGTTCACGAGCTCCGCGATGCCCCACTCAATCGACCA

CAATGTCGTCTCAATGGCAGCGACGTTGATGTTTTCTACAATGTAGAGGACGTTGTCTTC

ATTGATTTCTCCCTTGTCTTGGGCATCGAGGATGTGGTCGATGGCGCATTTTAGCCCAGA

TGCGCTGGCCGAGCCTTTGGTGCTTGACAGCTTCTTCCTCTCCTCAAGAAAGTAGTCCTT

GAAGAGCTGCAGCCTCCTTTCCTTCACTTCCTTGCAAATCTTCAAATACCCTCTCAAGAA

AGGCCTCAAGATGGGAATGAAATCACCGTAATTATAATCAAAGCTCTGGGCTAATCTGCT

CCGTTCACCATTCAATGCTTTCAGCTTCACGAACAGAGGATCTTCCTCACTCTCAAATCT

CCTATCGAACATGATCCGGTACATGTTGTTGTACATCATCAGCTGTAGCCTTCTCCTCAA

TACAATCCCCTTCGTTGCCGCATCTGGATTCGCTTTCACATCTTCCACCACACGTCCGAT

CTCGTCTTCCCATCCGTACCTGTACTGCTGGACCACCTTGTTCGTGAAGAACGGGACGGT

CATGATCCGACGCATCTTACGCCAGTGATCACCGTAGACCGTGAACACCATGTCCTGCCC

CTTGCCTGTGAAGATGTCGAAGACGACGTTTCGGGTCCTGGACCCGAACTCGACGCCCTG

AGTGTGGAGGACTTCCTTGGCGAGGTCAGGGGAGGAGACGACGACGAGGTTGCGCTGGCC

CATTCGGAGGAGTAAGATCTCTCCGAACCGACGGGCGAGATCGGACAGGTTTCGATGGTT

GAGATCATCGCCGACCTGGAGCCAGTTGCCGAAGACAGGGACGGGGATTGGGCCCGGCGG

GAGCTTGAATTTCTTCCCGCGTAGCTTTGAAACGACGGTGGCGACGATGATGGAGAAGAA

GAGAGCGAAAAGGGCCTTCTGCAAGAGAAGGAGATCCATCGCCGAGAATGAACGGCTGGA

ATTGAGAGAGAGGGAGGTGGGAGAAGGTGGTGCAGGTGAAGCGTGAGATCTCTG

3. comp43573_c0_seq2

GGGACACATAACTCGGTGCATCAAGAAGAGAGAAAGAGAGAATTAGGAAATGGGAGGAGA

GATGAAGGGACCAGTGGTGGTGACCGGAGCAGCTGGTTTCATCGGTTCGTGGCTGGTTAT

GAGGCTTCTTGAGAAGGGTTACACTGTCAGGGCCACCGTAAGAGACCCCACTAATATCAG

CAAGACCAAGCATTTGTTGGATTTACCTGATGCCGCCACTCGACTCACTATTTGGAAGGC

CGACCTTGATGATGAAGGAAGCTTTGACCATGCGATCGACAGGTGCACCGCAGTTTTCCA

TGTTGCTACCCCAATGGATTTCGAATCCACCGATCCTGAGAATGAAGTGATAAAGCCCAC

AATAAATGGAGTCCTAAACATCATGAGATCTTGCAAGAAGGCCACATCGGTTAAGCGCGT

CTTCTTCACGTCCTCCGCAGGGACAGTCAATGTCGAAGAGCAGCAGAAGCCAGTGTACAC

TGAAAGCAGCTGGAGTGACATCGATTTCTGCCGAAGAGTCAAGATGACTGGCTGGATGTA

CTTTGTCTCCAAATCACTAGCGGAGAAAGCTGCATGGGATTTTGCCAAAGAGAACAACAT

AGACTTCATTAGCGTCATACCGACCCTCGTGGTGGGGCCCTTCATCATGCCCAGCATGCC

CCCCAGCATGATCACCGCCCTGGCATTAATCACAGGAACTGAATCTCATTACTCAATTTT

AAAGCAAATCCAACTGGTTCACCTGGATGACCTATGCATGGCTCATATCTTCCTCATGGA

GCATCCAGAAGCTGAGGGCAGATACATCTGCTCTTCTCATGACACCACCATCATTGAACT

TGCAAAAAATCTCAAGGAAAGATACCCTGAATACAC

4. comp47103_c0_seq1

AGAGAGAGAGAGAGAGAGAGGAAACGAGTGATGGCTCCAGCTACGCCCTTCCTACCGACG

GCTACGAATGAGACGACACTACGATCTAGCTTTATCAGGGACGAGGATGAACGGCCTAAG

GTTGCTTACAATCAATTCAGCAACGAGATCCCCGTCATATCTCTGGCCGGCATCGACTCT

CCTGATGGACGGTCGGTAGTTTGCAAGAAGATCGTGGAAGCGTGCGAGGACTGGGGAATC

TTCCAGGTGGTGGACCACGGTGTCGATTCGGACCTCATTGCGGATATGACTCGTCTCGCT

CGGGAGTTCTTCACTCTCCCCGCTGAGGAGAAGCTCCGTTTCGATATGTCTGGTGGGAAG

AAGGGCGGTTTCATTGTCTCCAGCCACCTTCAGGGTGAGGCCGTACAAGACTGGCGGGAG

ATCGTAACATTCTTCTCATACCCGATCAGACTCAGAGACTACTCGAGGTGGCCCGACAAG

CCAGAGGGATGGATAGAGGCTGCTAAGAAGTACAGCGAACGCCTGATGGAGCTGGCATGC

AAGCTCTTGGAAGTACTGTCGGAGGCGATGGGGCTCGACAAGGAAGCTCTGACCAAGGCG

TGTGTCGACATGGACCAGAAGATGGTGGTGAACTTCTACCCGAAATGTCCCCAGCCCGAC

CTCACCCTCGGCCTGAAACGACACACCGACCCTGGCACCATCACGCTACTGCTGCAGGAC

CTGGTTGGAGGCCTCCAAGCCACCAAAGATGGTGGTAAGACCTGGATCACCGTCCAGCCC

ATCGAAGGAGCCTTTGTCGTTAACTTGGGTGATCATGGCCATTTTCTTAGCAATGGGAGG

TTCAAGAACGCCGATCACCAAGCAGTTGTGAACTCGAACTGCAGTCGCTTGTCCATCGCC

ACATTCCAGAACCCAGCTCCAGAGGCAATTGTGTACCCACTCGCGATACAGGAGGGAGTG

AAGCCAGTGATGGAAGAGCCAATCACTTTCGCGGAGATGTACCGACGAAAGATGAGCCGA

GATATAGAACTCGCCAATCTCAAGAAGCTTGCCAAGGAGCAGAAGCAAGGTGAGGAGCTT

GATAAGGTTACTGTCCAGAACCAAGCCAAGCCAATTGACTCGATCCTTGCTTAGACGGAA

GAAAAGCAGCAACTAGGTAGCAGTCGTGGCTGGATGACTCTGCTACAATAGTCTGGTCTC

TTTCTCTATCTTTTACAAATAAATATGTGGATCTCTATGTGGGTGGTTGTAATAATGGAT

ATATAGAGTTATGTAATGTTTCTTTTAAAAAAAGTAATTTGTTTTTCAGTTGTTTGTTGT

AGTTGAGATGATGATAATGATCAGTTGGAATGCAAGTGATTTTGGCTCCCTTTCATAATT

AAAAAAAAA

5. comp49650_c0_seq1

TTTTTTTTTTGGAAACACGAAGCAGAGAGTTCCGCATTTATTAATAATAATAATATAAGA

AGGGCAAAAGCAGCAGAAACAATCCATCCCTCAACTCTCTACTGAGGAAGTTTATTTAGC

TTACAATACTGATAATCCTTGTACTTCTTTGTCTTGAATTTAGGAGGGTTTCGGTCGTCC

ACAAACTGACTGAGAGGTCCTACCACCATTTCAGCTGGTGGTGAACAAAATACCGGCCAT

GACATCCTTGTCTTCTCCTTGTTTACTGTCGTCCTGTGCAACACGCTCTTGTACTTTCCA

TTACTTAATATCTCAATCTGGTCCCCGATGTGGACAATTAGAGCGTTGGGAATGTACTTG

ACGTCGAACCAGATATCGTCCTTGAAGACCTGCAGACCTGATATTTCATTAGGCACAAGA

ACAGTTATTGCTGACATGTCGGTGTGGGCCACCACACCCAACGCAAGATCTGGCCGAGGA

CATGGAGGGTAGTAATTTATCTTCAACAGAAACTCCAGATCATCTCCACCCACGGCGTCT

TTTAGCACACCCGCCTCCAATCCTAGCCCTAGCGAAAGGCTACTCAGCAGATCGTCCACC

ACATTCAGCATGTATTTCGCGTACTCCTCATTAGCCTCCCTGTAAGCAGGAGGGTTCTTG

GGCCACACATTGTAGTTAATGCGAGAAAGTGGCCATATGTTGTGGAAGAAGAAATCCACC

CAAGCCTTCTTACCTTCCATGTCCTTCTGGAGCTTTGTACCGTAACCTTCCAACGTATCG

GATTCCGGCTTTATTGCATACTCCTCCTTCTCTTCTTGTGGAAGCCCGAAGAATTCTTTC

CCTACATCTTGCAACTTTCTTATTATGTCGTCTGGTATCCCATGGTTTACTACTTGGAAA

ATTCCCCACTCTTGGCTAGCATCAGCGATTTCTCGTATGATCTTTTCGCGATTTGGGTCA

CTAAGATCGATGGTCGGGACATCAGGGACTGGACCTTGGAAGGTGGTGATCCCCGGACGT

TCGTTCTCTGAACGGACGAATTCGGCAGGGATGCCATCTTTGGCTGTGAACATAGATGCA

ATGGCCTGCACCCTTTCAACCTCCATTCCTAGAAAAATCAATTGAGTCAAAATGTAGTAG

AAAATGGTGTATG

6. comp50438_c0_seq1

TTTTTTTTTTTAAAATAAAAATACTATTATTTAGATAATAATAAGAGAATGACGTGGACA

TGGCTTCCAGGCCAATGTGTGGTCCAAGTCAACCAAAACCACAAACCAACACAACCACCG

TCCAACTCTCCATTTCACATTTCAACACCCACACGTATGGAAACAGCAAAACACCGCCAG

CAATCGAGCCAATGCATGCATGCATGCAGCCCTTCTTTCACACGTGCAATCTTATACTCT

TACTTAGCAGCCACACTGTGGAGTACGACGGTCTCGACAGTGAGCCCAGGCCCAAAACCA

AAGAGCACGCCCCACTCCAGCCCCTCGCCGGTGGTCACCAACCCATCCTCGGCCGACTTC

CGCCGCATCTCATCCAGTATAAAGAGCACGCATGCGCTCGACATGTTGCCGAACTCGCTC

AGGACGTGCCTCGTCGCTCGCAGCTTCTCGGCCTTAAGGCCCAGCTTAAGTTCCACCTGG

TCTAGGATCGCGGGTCCGCCAGGGTGCGCAATCCAGAAGAGAGAGTTCCAGTCTGAGATC

CCCAAAGGATCGAAGGCCTCAACCAGGCTTTTCTCTATGTTCTTCGATATGAGGCCCGGG

ACGTCTTTGAGGAGGTGGAAGGTCAGACCCACTTCCCTGAGGTGACCGTCGATGGCCCCA

TCGCTGTCCGGTAGGATTGTCTGGGCTGCACTGACCAACTCAAAGAGTGGGGACTCTACA

CCAGGAATGGGATCCGCACCGACAATCACAGCAGCTGCGCCGTCTCCGAAGAGCGCTTGG

CCGACCAGGCTATCGAGGTGGGTGTCGCTGGGCCCACGGAAGGTGACGGCAGTGATTTCG

GAGCAGACGACCAGGACACGTGCACCCTTGTTGTTCTCAGCTAGATCTTTGGCGAGGCGG

AGGACGGTCCCACCAGCGAAGCATCCCTGCTGGTACATCATGAAGCGCTTGACAGATGGA

CGGAGGCCGAGGAGCTTGGTGAGCTGGTAGTCAGCGCCTGGCATGTCGACGCCGCTGGTG

GTGCAGAAGACGAGGTGGGTGATCTTGGACTTGGGCTGGCCCCACTCCTTGATAGCCCTG

GCTGCAGCTTCCTTGCCTAGCTTGGGTACTTCAACCACAACCATGTCCTGTCTAGCGTCC

AAGGAAGGGGCCATATAAGCACACACGTTTGGGTTCTCCGTCAATATCTCCTCCGTTAGG

TACATGTACCTTTTCTTGATCATAGACTTTTCACACATGCGCTTGAACTTCTCCTTCAGC

TCTGTTTTGTGCTCGCTGTTGGTGATGCGAAAGTAGTAGTCCGGATACGTGTTTTGGTCC

ACCGCGTTGGGTGGAGTCGCCGTGCCGATCGCCATCACGGTGGCCGGGCCTTCGGCTCTC

TGCTCCTTACGGATGACGTCCAAACTCACCATCTCGGCCGATTGCTGGATCTTACACGTG

GTAGTGTTGGATGCACGTGCCTGTGTTTGTGTGGTGGGGAGTGGTAATGAATGGTCATCC

GTAAGGAGCAGA

7. comp51086_c0_seq1

TGGCCATTCCCTCTCAATCTTTCTTCACAAACACTAATAGAAAAAACAAAAAACAGAGAG

AGAATGGCCACACAAGTAGCTTCTATCCCCAGAGTGGAGATGCTTGCGAGTGCAGGCATC

CAAGCAATACCGACGGAGTACGTCCGTCCTGAGGCAGAGCGGAACAGCATCGGCGATGTG

TTCGAGGAGGAGAAGAAGTTGGAGGGACCCCAAATCCCTGTCGTTGATTTGATGGGCTTG

GAATGGGAGAACGAGGAGGTTTTCAAGAAAGTGGAGGAGGATATGAAGAAGGCGGCGTCG

GAATGGGGTGTGATGCATATCATCAACCATGGGATCTCTATGGAGCTCATGGACCGTGTT

CGGATTGCTGGGAAAGCGTTTTTCGATCTGCCAATCGAGGAGAAGGAGATGTATGCCAAC

GATCAGGCTTCAGGGAAGATTGCTGGCTATGGAAGTAAGCTTGCTAACAATGCAAGCGGG

CAGCTTGAGTGGGAGGATTATTTCTTCCATCTCATCTTTCCTGAAGATAAGCGAGATATG

TCTATCTGGCCTAAGCAGCCCAGCGACTACGTCGAGGCAACCGAAGAATTCGCAAAGCAG

CTGAGAGGACTGGTAACCAAAGTGCTAGTACTGCTGTCTCGCGGATTAGGAGTAGAAGAA

GACCGTCTCGAGAAGGAGTTCGGCGGCATGGAGGAGTTGTTACTACAGATGAAAATCAAC

TACTACCCAAAATGCCCTCAGCCAGACCTAGCTCTAGGCGTGGAGGCCCACACCGACGTG

AGTGCACTCACCTTCATACTCCACAACATGGTCCCAGGCCTTCAGGTCTTCTTCGACGAC

AAGTGGGTGACGGCCAAGTGCATCCCTGGCGCGCTCGTGGTCCATATCGGCGACAGTCTG

GAGATCTTAAGCAATGGAAAGTACAGGAGCATCCTCCATAGGGGCCTCGTCAATAAGGAG

AAGGTCAGGATTTCCTGGGCCATTTTCTGCGAGCCTCCTAAAGAGAAGGTCGTCCTCCAG

CCACTGCCAGAGCTGGTTTCTGAGGCTGAGCCTGCACGCTTCACCCCTCGTACGTTCTCT

CAGCACGTGCGCCAAAAGCTGTTCAAGAAGCAGCAAGACGCTCTCGAAAACCTCAAATCC

GAATAAGAGAGTCATTGCTCTCTCTCTCTCT

8. comp25552_c0_seq1

GCCTCACATAACCTTTCTCAACAATAACAATCTGCACCCATGAGAGCCCAGAGCCCCAGA

AAAAGCTCGAGAAATACAGAAAGCCTAAGAATACTCAATCAAAGACATACACAGAGTAAT

ATTTAAAAAATAAAGTAAAAACCAAGATAGGTTTTTCTCATATTACTGATTATTCACTGT

AAATTCCAATTACAAACAGACCCAGATGAGATTTCAATCACAAGAGCTCGAATTTCCACA

GCTCCTGATCAAATCCCATCCTCAGATACTGGCTGAAAGGGTCAACAGGTGGTTTCCTGT

CCCCATCCTCACAAATTAAATCCCAACCGTTGAGTTCATCAACACCCCACAGACACATGG

AATCCGACCACTCCATGCTGGGAAAATCAAATCCGTCCGGTCTCCAAGAAGAATCAGATG

AAGTAGATGAATCAGATGTAGTAGCACATGGCACTAAAATCTCATGAGGCTGGATCAAAG

GTACCTCATCAGTGCAAAAACCAGGGGATTTTCTTAAAAGATCAGCACCAGCTTCAATGG

AAGTACTCAATGCATTTGGAATGCTCTTTTCTTCTTCTGTTATATCAGAAATGGGTGATT

GAGAGGATGAATTGCTGTCCTTTGTTTCATCTGTATTATCAAAAGCAACTACAGCAGATG

TTTGTTGTGGTTGTTGTTGTTGT

9. comp45264_c0_seq2

TCACAAACGACTGTATATTTAAAATCGTCTGCTGGCTTCACAGGAAAAACAGAACACGGG

CTGTTATTCATGGGTCAATCAGGAGTTCGAAAGGGTGCATGGATCAAAGAAGAAGATCTT

CTGCTAAGGAAATGCATAGAGAAGTATGGAGAAGGGAAATGGTATCTAGTTCCTCAACGA

GCAGGCCTCAATCGGTGTCGTAAGAGCTGCAGATTGAGATGGTTTAACTATCTCAGGCCA

AATATTAAGCGAGGAGAGTTTGGTGCAGATGAAATTGATCTCATCAGTAGGCTTCATAAA

CTATTGGGTAATAGATGGACGTTGATTGCAGGTAGAATTCCAGGCAGGACTGCCAATGAC

ATAAAGAACTTTTGGAATTCACACTTCAGTAAAAAAGTTACTTTCCATTACGACGGGAAG

ACTGCAAAAGCTAGCAATGCCAAAGTCATAAAGCCACAGCCCAGGAAACTCTCCAAGAGT

TTGCCTAGCTCTGAGAATCTTGAAGCCTCGAGAATCCAAGAACAGTGTGGCAAACCACTA

TTGGCTCCATACAAGTATTCAATGTGGTTGAATGGTTTGTTCCAAGAGGGAAGAGAAAAT

TGGCCTTTGAACAATGGAGATGCAATGGGAGAACTGTTACTTCCTACCATTGACAATGGA

GAGGCTGAAGGAATAAGAGATGAAGATGTAACTTTTGCCGAAGACCATCAAGATTGGGAT

GACTTGATTTTCGATCCAAGTCTTTGGGCGCAATTAGATGGCCACTGATTGGTTGACATT

TTAAAATCGTTTGATCCATGTTAGTATTAGGGACTGCTTGTTTTTTTGATTTTCTGTGTA

ATTACAAGTAAACAATCTATTATTATCATTAGAAGGTGTTTGTTAACATGAATGCATTTA

GG

10. comp45298_c0_seq2

GAGAGAGAGAGAGAGAGAGTTCATGTCCACACAATCTAATACATAACAGTTTATCAACAA

CGAGGAAAAAGATGAATTTATAGCATACATATAAATTACCACAAATCTCTAATTTATTTA

GTGCCGATACCATCTGCAGATCATTTATAACTGCCCATCAACCCGATCATCATCTCGCCT

GCAGTAGTCTTCGTTTACACCTGCAGGAGACAAAACTGCAGCTTCCCAATAAGTGTGGAA

GCCGAAACTCGGCGTCGAAGGAACCAAATGATCCAACGGTTCGGCCACAATCTGCTGCTG

TAAAAACATGTCTTCATTGATTAATGAATTCAAGAACGAAGAGAATACATCCTCGGTGCA

GCAACCCATATCATCATCTTCAAGACTCATGCCTCCTGGCACATCGTAATTCCGCCAATT

CTCATCTACGTCCTCGTCGCGCAAGATCCAACCCCCACCATCCCCATCCAGGTTGGTAGT

CCCATCTTTATGTTCAGAATCCTCACCTCCTCCCTGTGAACCGGAAGAATTAGGGTTAGG

GTTGGGTTCGGTAGATGGTGGTGCATGGATATTAGGGTCAGGGTTCATGGGCTTATGTGT

CCGCGGATCGATCCCTTGACTGATCAGCTTCTTGCTAAGGTGGGTATTCCAGTAGTTCTT

GATCTCATTATCCGTACGGCCTGGAATCCTCCCAGCTATCAGAGACCACCGGTTTCCGAG

TAGTCGGTGCAAGCGTAGGATTAGATCTTCCTCGTCAGGTGCGATCTGGCCGCGCTTGAC

GGACGGGCGGAGGTAGTTCATCCACCGTAGGCGGCAGCTCTTGCCACATCGGAGTAGGCC

TGCTCGCTTCGGTAGCGTCCGCCACCGGCCCTCTCCCTCCCGGGCTATGTAGTTGCAGAG

TAGCGAGTCTTCTTCGGGCGTCCATGGGCCCCTCTTCAGGCCCACCTTGCTACAGCAAGG

TGTGGTCTTGCAGGCCATGGCCGACGCAGTCGATGATGATGTTGCTGGCGGGCTTCTCAT

CTTCTCTTCGTTCTCTTTTTCTGCATTTTTTGGTCTGAGGATGCGAGCAGCGATATATAT

GGGTGCACAGGAGTTCGTTTTACCTCTGATGAGACTCAGGATCTAGCGTGTGTCGCCCAC

TTGGGGGCTACATGCTGAGATGGTACGATGATCTGAACCGTCCATATTATTTTCAGTACC

ATGATAAGGTATTTTTTTT

11. comp46821_c0_seq4

TTTCCTCTCTTTCCCATCTGACTCTCTTTCTCACCATAACATATACTACAATCTCTCTTT

CTCTCTCTCCACATGGGTCGTTCTCCTTGCTGTGAGAAAGCCCATACCAACAAAGGTGCA

TGGACCAAAGAGGAAGACCAACGCCTCATCTCCTACATCAAAGCTCATGGAGAAGGTTGT

TGGAGATCTCTCCCAAAAGCTGCTGGGTTGTTGAGGTGTGGGAAGAGTTGCAGGCTCAGA

TGGATAAACTACCTTAGACCTGATCTCAAAAGAGGCAACTTTACAGACGAAGAAGACGAG

CTTATCATCAAACTCCACAGCCTGCTTGGGAACAAATGGTCTCTCATAGCCGGACGCCTG

CCTGGGAGAACCGACAATGAAATCAAGAACTATTGGAACACACACATAAAACGGAAGCTC

CTTAGCCGAGGGCTTGATCCTCAGACCCACCGTCCGATCGGCGAATCATACTCCGACATG

TTAAATTCCACACCTCAAAGAGACTCAACATCACCTGAAATCGCCATCTCCTACACAAAT

AATAACAAAGGAAATAGCCCCAATCCCATCGATTCTCAAGACGAGGCGAACAGCAGCAGC

GTGAGTGAAGAAGTGCAACACTACCCAGAACTCAACCTCGAACTCTCAATCAGCCTTCCC

TTCCAATCCAATCCGCCTTCAGCAAATACCGCAGAGTCAAAGCAGCAATCCTCATTCAAT

TCGGCCTTAACGGCCGCACGGACGCCACAGCCTTTCTTCACTACGGCCGTCTGTTTGTGT

TGCCATTTAGGGTTTCAGAGCAGTCAAGCCTGTCGTTGCCAGACAATCCCAAACGCTCAA

GCACTCAGATAATACACACCTGTGCATTCTTAGTCCCTTGTAATAATACCTCCTTGATAT

ATCTTAGTGAATTTTTGACAGAAGAATGTTGTTGTTCATGGTGGTTGTTTCCACGGTACA

TTACGATAGCATTGTTTTGAATTTGTGATGTGAAAGTATTTACAAGTGGGTTCATGTTCA

AATATCTGATGGACCCCATCACAGATGGGAGATGCCCA

12. comp47225_c0_seq1

TTCCTCATCCATCCTTCAAGCAATTGGGATACAGTATCTGTGCTCCATGCGTAGGTAGAT

GGTTGGAGCGGTCTGGTGTGGGATTGAGAGCCATGGGTAGCCTTTGGGTCCAAAAGAAGG

TCTGATTTCTCAATGGACAAGGCCTCACATAGAGCTTGCTTGGCCATGTGAATGTCAGTT

TGGAGCCTTCTCTCCCATTGTCCTTTGGAGATAGGCTGTGTGGCTGAGGTCCCATTGCTC

AGATGGCCATCCACACATGTTTGGAGCTTCTTAAGCTTTTTCTTCAAGTAGGTATTCCAG

TAGTTCTTGATATCATTGTCTGTCCTTTGAGGTAGGTAAGAAGCTATGGCAGCCCATCTA

TTGCCCAAGAGAGCTTGAAGGTGGATGATCTTCTCCTCCTCAGTATCAGTGAAGTTGCCA

CGTTTGATCCCGGGCCTAAGGTAGTTAGTCCACCTAAGCCTGCAGCTCTTGCTACATCTA

AGCAACCCTGTGTTAGTAGGAACAGCCCTCCAATTCCCAGCACCATGTTCTTGAATGTAA

GAGACCAAGATAATATCTTCTTCAGGTGTCCAAGGGCCCTTCTTCATACCCATTTTGTCA

CAGCAAGGTGGTCGACCCATGATGCCTTTTCTTCTTCTTCTTGGGTTTTCTTTCTTATTT

CTCTCTAAGGGTGTTTTTTTTTTTTTTTTTTCAAGGGAG

13. comp47225_c1_seq1

GCTTGAAGGATGGATGAGGAATTCCCCGAAGAAGATGCGACAAACGGCGTTCGAGATGAA

TCGTAATTCATTCAACAAAGGGGTCGGAGCAAATTCCAACTCCAGCGAAGAGACGCCAAG

GACTGTGAAGAATGGAGTTATGTCATCAGAGGCTCTTGAATCGCTGCTCAGCTTTGATGA

GTCTTCGATGACGCCGGACACATCTCAGTCAGTGTCTCTAGATGAGAATGCGAATTATGC

AGCTGATATGGGCTTTTTCCAAGCTGAAAGCAAGCCCAATTCAGAGGCCCAAGGCCCACT

TTCCGTGCTTGAGAAATGGTTGTTGGGACACGATGACATGATAGAGATGCCAGTAGATGA

GACTGCTGACTTGTTTTGAAGGGTTTTTCTTGTTTTGGGTCTTTGGATATTCTTTCCATC

TTTGATTTGGGTTTTGATTCTTTGTTTTGTTACAAGGCCTTGAACTGATTGAAGATATCT

CTTGTTTGAGCTTATCAGAAAGACATTCCCAAATCTAAGAAAAGAG

14. comp47747_c0_seq2

GAGAGATTTAAAGTTAGGATTGAAACTTTACTGGATTTTGAAATTCTAATAAAAAAACAT

CAGTTTATGTAGTTTCTCTGTTTTACTCAAATATCCAATTCAAAAAAATAAAATAAAATA

TAAAAATAAAAATATCATTCTTCTCTTCCATCTGTACACTTAAATCTACCCAAAGATTAT

TAGGTATCGAAGAAGAATGATGATTCATCTCCTAATTTCACCTCCTAATTTCACTACTCC

GTTTTCGGAACTCCGATCCACTTCACCAATGGATCATTCATACTCTCTGCTGCTTTAAAA

CAATATCTACTCTTTTGAGTCATACCCAACATATAACTCTTCACTTCTTCTCTAATCATT

TCCTGCATCACTAATACAAACTCCTTACTCAAAGCAAATTGCCTATTTCCAAGATTTTCA

GCTGCACTGTTTTGGGCAATCTGTTCGCTAGGGCTTGATTTGGGAGACGATCTTGAGTCA

TCAGGCGATCCGGTCCAAAGAGATAGGGATAGGTCAACAGATGTTGATGGAATGGTATTT

GGGGCAGGGGATGAGATCTCAACTTGCAGGGATGGTGGCTGGACTGGTCCGGTTCTCGCC

ACAGGTCGGTAGACGTTAGAAGAAGAAGCCAGCCGTTGAAGGCTAAAATCGCTTAGATCC

GATCCGGATGTGCTGCTAGGCCCGGCTCCGGCGCTCATCGACCGCTTCGCCGGTGGCTCA

TCTGGAATAGCGTCGAGATCCGCTGCCGCATCTTCAGATGATGCCGGACGGCATTTTCTC

TTGAGCGTTGAATTCCAATGGTTTTTGATGGAGTTGTCGGTGCGGCCGGTGAGAAGACGA

GCGATCGTGGCCCACTTGTTGCCGAGCCTGTCATGAGCCTGGACTATGATCTTGTCTTCC

TCGGCAGTAAACGGACGGTGCGCGACCTGCGGCGAGAGCTGATTACACCATCTGAGGCGG

CAGGACTTGCCGGATCGGCCTGGGATGGATTTACTGATCAGGGTCCAGTTCCGCTGGCCA

TGCTTGCTTACGAGCTTCTGGAGGCATTGATCTTCTTCCGGACTCCATGGACCTTTGATC

CGTTGTAGATCTGAAGGAGAGGCCATATAGACGATTTAGATCTCTTTGATCTAATGACAG

AACTAGAAACCCTAACCCTAGAATTCCAGTTCTAGGAAATTTGGAAAGAGGCCCGTGGAG

AGGCGCGGAGTTCAATGCCTCCAGG

15. comp50161_c1_seq1

CACTGGGAAGAGCTGCCGGCTCCGTTGGCTCAATTACCTTAGGCCTGACGTCCGGCGGGG

GAACATCACACCAGAAGAACAGCTCTTGATCATGGAGCTTCATGCTAGGTGGGGAAACAG

GTGGTCGAAAATTGCGAAGCATCTTCCGGGAAGAACCGATAATGAAATAAAGAATTACTG

GAGAACAAGGATCCAGAAGCAAATTAAGCAGGCTGAACCTTTCGCCAACCAGAATTACAC

TCTCGCACACGATGCGAGCACAAGTCATGCTTGTGGTGCCAACGACGAAGTTGATCCGTA

TTCGCCAGCATACACCATGAGCAATCCTGAGCCGTTTGCAACTCCTTTGCCCACCGAATC

CAACGATTGCTTGTGGAGCATGGAGGATCTCTGGTTCCCCCAGCTGTTTAATGAAGATTA

AATAAAATTGGCTTTTCATTGTTCTCCATAATGATTTTAGTTGTATAACCTAGTAATTGT

GGTGTGTGGTACTACAGAAGAACGCTTAATTAAGTATAGATTTGTTATAAATGAAGATGT

TTTACTTATTTACATTCCACCTTAATATTGCTCAGAGAAACCACACATCTGTGCGATTGC

AAGCTATTTGAAAAGATTGGAAATCGATACTTCAAATCCCCGGTCACAGACGAACAACTA

ATTTAGGTAAAACCCGAATCCAGTCCTAGTGTTACGTCATATAAAGCTGATGATTTTTTA

CTTAATTAAGTACATTATGCTTCTATGATTCGAGCATGTTTTTCAATGATCATGATGTGA

TGGTGGACACTTTCAAGTGAAAGAATGGTGTCGTCTCTCTTTTGCTTTATCACAAAACCA

TGACATATATATAAGTAGAATTATTTGTACTTAATC

16. >comp53953_c1_seq1 (Actin)

GCTAAAATCTGGTTCCACGAGACAGCTGGTTACACGAGAGACGCTTAATCCAGGCCGCTG

GTCGAATAGCCGCAACTAACGGTGGAGATTAGATCTTATGAATCCGAGGTTCATTTCTCC

TGGGTTGGAGTTTCATTTGGGCGAGATTTCCTTTTAAATCCAAGGGCGGGTAGTTCATTA

CCAGAAAATCTCTCAGAGAGAGAGGGGAGGGAGAGAGAGATCTAGGGTTTGAAAGGTACT

AGAAGATGGCTGATGCTGAGGATATTCAACCCCTTGTGTGCGATAATGGAACTGGAATGG

TGAAGGCTGGATTTGCTGGAGACGATGCTCCAAGGGCTGTGTTTCCCAGCATTGTTGGCC

GCCCACGTCACACTGGTGTAATGGTTGGCATGGGGCAGAAGGATGCCTATGTTGGGGATG

AAGCTCAATCCAAGCGTGGTATTTTAACCCTGAAATACCCAATTGAGCATGGTATTGTGA

ACAATTGGGATGACATGGAGAAGATCTGGCATCACACTTTCTATAATGAGCTACGGGTGG

CCCCTGAAGAACACCCAGTTCTCCTCACCGAAGCACCACTCAATCCCAAGGCTAATCGTG

AGAAGATGACACAAATCATGTTTGAAACCTTCAACACTCCTGCTATGTATGTTGCCATCC

AGGCTGTTCTGTCCCTTTATGCCAGTGGTCGCACGACTGGTATTGTGCTGGACTCTGGGG

ATGGTGTTAGCCACACGGTCCCCATCTATGAGGGATACGCACTCCCACATGCCATCCTGC

GTCTCGACCTGGCAGGCCGTGACCTGACAGATGCTCTTATGAAAATACTGACAGAACGTG

GCTACTCTTTCACAACCACAGCTGAGCGTGAAATTGTGAGAGACATGAAGGAGAAGCTGG

CCTACATAGCGCTCGACTATGAGCAGGAGCTAGAGACATCCAAGACCAGCTCTTCTGTGG

AGAAGAGCTATGAGCTACCTGATGGGCAGGTTATCACCATTGGGGCTGAGCGTTTCCGTT

GCCCGGAGGTGCTTTTCCAGCCTTCAATGATTGGAATGGAGTCTTCGGGCATACACGAGA

CCACATACAACTCCATCATGAAGTGTGATGTCGATATTAGGAAGG
