# Supplementary material for: Genome-wide transcriptome analysis of genes involved in flavonoid biosynthesis between red and white strains of Magnolia sprengeri pamp
Source: BMC Genomics. 2014 Aug 23;15(1):706. doi: 10.1186/1471-2164-15-706 (PMC4156625; doi:10.1186/1471-2164-15-706)

Additional file 2: Anthocyanidin analysis of petals in red and white flower color. Peaks of HPLC were identified by retention time compared with cyaniding-3-O-glucoside chloride standards. (A: Red petals; B: White petals)


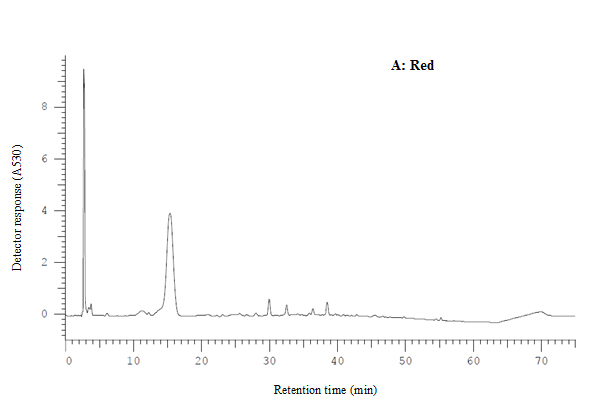

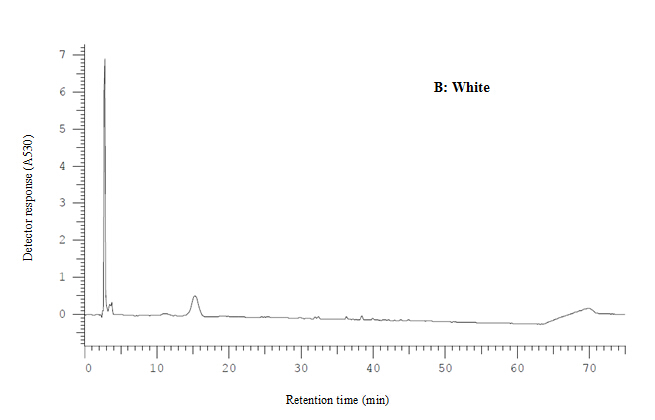

Supplement: Supplementary file 2 — Additional file 2: Anthocyanidin analysis of petals in red and white flower color. Peaks of HPLC were identified by retention time compared with cyaniding-3-O-glucoside chloride standards. (A: Red petals; B: White petals). (DOC 124 KB) [file 12864_2014_6403_MOESM2_ESM.doc]
